# COVIDENZA - A prospective, multicenter, randomized PHASE II clinical trial of enzalutamide treatment to decrease the morbidity in patients with Corona virus disease 2019 (COVID-19): a structured summary of a study protocol for a randomised controlled trial

**DOI:** 10.1186/s13063-021-05137-4

**Published:** 2021-03-16

**Authors:** Karin Welén, Anna K Överby, Clas Ahlm, Eva Freyhult, David Robinsson, Anna Jonsson Henningsson, Johan Stranne, Daniel Bremell, Martin Angelin, Elisabeth Lindquist, Robert Buckland, Camilla Thellenberg Carlsson, Karlis Pauksens, Anna Bill-Axelsson, Olof Akre, Cecilia Ryden, Magnus Wagenius, Anders Bjartell, Anna C. Nilsson, Johan Styrke, Johanna Repo, Åse Östholm Balkhed, Katarina Niward, Magnus Gisslén, Andreas Josefsson

**Affiliations:** 1 Department of Urology/Sahlgrenska Center for Cancer Research, Institute of Clinical Sciences, Sahlgrenska Academy, University of Gothenburg, 405 30 Gothenburg, Sweden; 2 Department of Clinical Microbiology, Section of Virology, Umeå University, Umeå, Sweden; 3 Molecular Infection Medicine Sweden, Umeå University, Umeå, Sweden; 4 Department of Clinical Microbiology, Section of Infection and Immunology, Umeå University, Umeå, Sweden; 5 Department of Medical Sciences, National Bioinformatics Infrastructure Sweden, Science for Life Laboratory, Uppsala University, Uppsala, Sweden; 6 Department of Urology, Region of Jönköping, Jönköping, Sweden; 7 Department of Biomedical and Clinical Sciences, Linköping University, Linköping, Sweden; 8 Department of Clinical Microbiology, Region Jönköping County, Jönköping, Sweden; 9 Department of Infectious Diseases, Institute of Biomedicine, Sahlgrenska Academy, University of Gothenburg, Gothenburg, Sweden; 10 Department of Surgical and Perioperative Sciences, Urology & Andrology, Umeå University, 901 87 Umeå, Sweden; 11 Wallenberg Center for Molecular Medicine, Umeå University, Umeå, Sweden; 12 Department of Radiation Sciences, Oncology, Umeå University, Umeå, Sweden; 13 Department of Infectious Diseases, Uppsala University Hospital, Uppsala, Sweden; 14 Department of Surgical Sciences, Uppsala University, Uppsala, Sweden; 15 Department of Molecular Medicine and Surgery, Karolinska Institutet, Stockholm, Sweden; 16 Division of Infection Medicine, Department of Clinical Sciences, Lund University, Lund, Sweden; 17 Division of Urological Cancers, Department of Translational Medicine, Lund University, Malmö, Sweden; 18 Department of Translational Medicine, Infectious Diseases Research Unit, Lund University, Malmö, Sweden; 19 Department of Infectious Diseases, Sahlgrenska University Hospital, Region Västra Götaland, Gothenburg, Sweden

**Keywords:** COVID-19, Randomised controlled trial, multicentre, protocol, enzalutamide, androgen signalling, TMPRSS2, antiandrogen

## Abstract

**Objectives:**

The main goal of the COVIDENZA trial is to evaluate if inhibition of testosterone signalling by enzalutamide can improve the outcome of patients hospitalised for COVID-19. The hypothesis is based on the observation that the majority of patients in need of intensive care are male, and the connection between androgen receptor signalling and expression of TMPRSS2, an enzyme important for SARS-CoV-2 host cell internalization.

**Trial design:**

Hospitalised COVID-19 patients will be randomised (2:1) to enzalutamide plus standard of care vs. standard of care designed to identify superiority.

**Participants:**

Included participants, men or women above 50 years of age, must be hospitalised for PCR confirmed COVID-19 symptoms and not in need of immediate mechanical ventilation. Major exclusion criteria are breast-feeding or pregnant women, hormonal treatment for prostate or breast cancer, treatment with immunosuppressive drugs, current symptomatic unstable cardiovascular disease (see Additional file 1 for further details). The trial is registered at Umeå University Hospital, Region Västerbotten, Sweden and 8 hospitals are approved for inclusion in Sweden.

**Intervention and comparator:**

Patients randomised to the treatment arm will be treated orally with 160 mg (4x40 mg) enzalutamide (Xtandi®) daily, for five consecutive days. The study is not placebo controlled. The comparator is standard of care treatment for patients hospitalised with COVID-19.

**Main outcomes:**

The primary endpoints of the study are (time to) need of mechanical ventilation or discharge from hospital as assessed by a clinical 7-point ordinal scale (up to 30 days after inclusion).

**Randomisation:**

Randomisation was stratified by center and sex. Each strata was randomized separately with block size six with a 2:1 allocation ratio (enzalutamide + “standard of care”: “standard of care”). The randomisation list, with consecutive subject numbers, was generated by an independent statistician using the PROC PLAN procedure of SAS version 9.4 software (SAS Institute, Inc, Cary, North Carolina)

**Blinding (masking):**

This is an open-label trial.

**Numbers to be randomised (sample size):**

The trial is designed to have three phases. The first, an exploration phase of 45 participants (30 treatment and 15 control) will focus on safety and includes a more extensive laboratory assessment as well as more frequent safety evaluation. The second prolongation phase, includes the first 100 participants followed by an interim analysis to define the power of the study. The third phase is the continuation of the study up to maximum 600 participants included in total.

**Trial Status:**

The current protocol version is COVIDENZA v2.0 as of September 10, 2020. Recruitment started July 29, 2020 and is presently in safety pause after the first exploration phase. Recruitment is anticipated to be complete by 31 December 2021.

**Trial registration:**

Eudract number 2020-002027-10

ClinicalTrials.gov Identifier: NCT04475601, registered June 8, 2020

**Full protocol:**

The full protocol is attached as an additional file, accessible from the Trials website (Additional file 1). In the interest in expediting dissemination of this material, the familiar formatting has been eliminated; this Letter serves as a summary of the key elements of the full protocol.

**Supplementary Information:**

The online version contains supplementary material available at 10.1186/s13063-021-05137-4.

## Supplementary Information

**Additional file 1:.** Full study protocol.

## Data Availability

The trial board will have access to the final trial and no contractual agreements limit the access to the dataset.

