# Supplementary material for: COVIDENZA - A prospective, multicenter, randomized PHASE II clinical trial of enzalutamide treatment to decrease the morbidity in patients with Corona virus disease 2019 (COVID-19): a structured summary of a study protocol for a randomised controlled trial
Source: Trials. 2021 Mar 16;22:209. doi: 10.1186/s13063-021-05137-4 (PMC7961321; doi:10.1186/s13063-021-05137-4)
Supplement: Supplementary file 1 — Additional file 1:. Full study protocol. [file 13063_2021_5137_MOESM1_ESM.docx]

CLINICAL STUDY PROTOCOL

| A prospective, multicenter, randomized PHASE II clinical trial of enzalutamide treatment to decrease the morbidity in patients with Corona virus disease 2019 (COVID-19). THE COVIDENZA TRIAL  Study code: COVIDENZA  EudraCT number: 2020-002027-10.  Ethical Approval: 2020-05-13 (Dnr 2020-02122)  Version number: Version 1.0 2020-04-22  Version 1.1 2020-05-27  Version 1.2 2020-08-20  Version 2.0 2020-09-08  Date: 2020-09-08  Sponsor: MD Andreas Josefsson  Norrlands universitetssjukhus, Umeå  Principal Investigators: Assistant Professor Andreas Josefsson | |
| --- | --- |
|  |  |

Table Of Contents

[A prospective, multicenter, randomized PHASE II clinical trial of enzalutamide treatment to decrease the morbidity in patients with Corona virus disease 2019 (COVID-19). 1](#_Toc38561835)

[Signature page 6](#_Toc38561836)

[Contact information 7](#_Toc38561837)

[List of used acronyms and abbreviations 9](#_Toc38561838)

[1. Synopsis 10](#_Toc38561839)

[A prospective, multicenter, randomized PHASE II clinical trial of enzalutamide treatment to decrease the morbidity in patients with Corona virus disease 2019 (COVID-19). 10](#_Toc38561840)

[2. Background and rationale 13](#_Toc38561841)

[SUMMARY 13](#_Toc38561842)

[Background 13](#_Toc38561843)

[3. Benefit-risk evaluation 14](#_Toc38561844)

[Potential benefits for study participants 14](#_Toc38561845)

[Potential risks for study participants due to enzalutamide treatment 15](#_Toc38561846)

[Equipoise 16](#_Toc38561847)

[4. Study objectives 17](#_Toc38561848)

[4.1. Primary objective 17](#_Toc38561849)

[4.2. Secondary objective(s) 17](#_Toc38561850)

[4.3. Primary endpoint (variable) 17](#_Toc38561851)

[4.4. Secondary endpoint (variables) 17](#_Toc38561852)

[5. Study design and procedures 18](#_Toc38561853)

[5.1. Overall study design 18](#_Toc38561854)

[Treatment arms 18](#_Toc38561855)

[Treatment duration 18](#_Toc38561856)

[Rationale for the choice of the patient cohort 19](#_Toc38561857)

[Explorative phase: 19](#_Toc38561858)

[Prolongation phase: 19](#_Toc38561859)

[Full trial: 20](#_Toc38561860)

[5.2. Procedures and flow chart 20](#_Toc38561861)

[5.3. Biological sampling procedures 20](#_Toc38561862)

[5.3.1. Handling, storage, and destruction of biological samples 20](#_Toc38561863)

[5.3.2. Total volume of blood per subject 20](#_Toc38561864)

[5.3.3. Biobank 21](#_Toc38561865)

[5.4. End of Study 21](#_Toc38561866)

[6. Subject selection 21](#_Toc38561867)

[6.1. Inclusion criteria 21](#_Toc38561868)

[Definition of COVID-19 positive test 22](#_Toc38561869)

[Evaluation of Severity of COVID-19 disease 22](#_Toc38561870)

[Mild 22](#_Toc38561871)

[Moderate 22](#_Toc38561872)

[Severe 22](#_Toc38561873)

[Evaluation of comorbidity at day 0 22](#_Toc38561874)

[Other diagnoses are listed in the day 0 form. Medical treatments for any conditions are listed in the day 0 form. 22](#_Toc38561875)

[Evaluation of other risk factors at day 0 22](#_Toc38561876)

[6.2. Exclusion criteria 22](#_Toc38561877)

[Subjects must not be included in the study if any of the following criteria are met: 22](#_Toc38561878)

[6.3. Screening 23](#_Toc38561879)

[6.4. Withdrawal criteria 23](#_Toc38561880)

[7. Study treatments 23](#_Toc38561881)

[7.1. Description of investigational product(s) 23](#_Toc38561882)

[7.2. Dose and administration 23](#_Toc38561883)

[7.3. Packaging, labeling, and handling of investigational products(s) 23](#_Toc38561884)

[7.4. Drug accountability and treatment compliance 23](#_Toc38561885)

[7.5. Randomization 24](#_Toc38561886)

[7.6. Concomitant medications 24](#_Toc38561887)

[7.7. Destruction 24](#_Toc38561888)

[7.8. Treatment after study end 24](#_Toc38561889)

[8. Methods for measurement of endpoints for clinical efficacy and safety 25](#_Toc38561890)

[8.1. Methods for measurement of endpoints for clinical efficacy 25](#_Toc38561891)

[8.1.1. Primary endpoint (variable) 25](#_Toc38561892)

[8.1.2. Secondary endpoints (variables) 25](#_Toc38561893)

[8.2. Methods for measurement of endpoints (variables) for clinical safety 26](#_Toc38561894)

[9. Handling of Adverse Events 27](#_Toc38561895)

[9.1. Definitions 27](#_Toc38561896)

[9.1.1. Adverse Event (AE) 27](#_Toc38561897)

[9.1.2. Adverse Reaction (AR) 27](#_Toc38561898)

[9.1.3. Serious Adverse Event (SAE) 27](#_Toc38561899)

[9.1.4. Suspected Unexpected Serious Adverse Reaction (SUSAR) 27](#_Toc38561900)

[9.2. Assessment of Adverse Events 27](#_Toc38561901)

[9.2.1. Assessment of causal relationship 27](#_Toc38561902)

[9.2.2. Assessment of intensity 28](#_Toc38561903)

[9.2.3. Assessment of seriousness 28](#_Toc38561904)

[9.3. Reporting and registration of Adverse Events (AE) 28](#_Toc38561905)

[9.3.1. Reporting of Serious Adverse Events (SAE) 29](#_Toc38561906)

[9.3.2. Reporting of Suspected Unexpected Serious Adverse Reactions (SUSAR) 29](#_Toc38561907)

[9.4. Follow-up of Adverse Events 29](#_Toc38561908)

[9.5. Independent Data Safety and Monitoring Committee (DSMB) 30](#_Toc38561909)

[9.6. Annual Safety Report (Development Safety Update Report, DSUR) 30](#_Toc38561910)

[9.7. Procedures in case of emergencies, overdose or pregnancy 30](#_Toc38561911)

[9.8. Reference Safety Information 30](#_Toc38561912)

[Enzalutamide safety information in summary 30](#_Toc38561913)

[10. Statistics 31](#_Toc38561914)

[10.1. Analysis population 31](#_Toc38561915)

[10.2. Statistical analyses 31](#_Toc38561916)

[10.2.1 Statistical methods 31](#_Toc38561917)

[Summary statistics 31](#_Toc38561918)

[Primary objective 31](#_Toc38561919)

[Seconday objectives **Error! Bookmark not defined.**](#_Toc38561920)

[Drop-outs 32](#_Toc38561921)

[10.3. Adjustment of significance and confidence interval 32](#_Toc38561922)

[10.4. Sample size calculations 32](#_Toc38561923)

[10.5. Interim analysis (if relevant) 32](#_Toc38561924)

[11. Quality Control and Quality Assurance 33](#_Toc38561925)

[11.1. Quality Assurance and Sponsor oversight 33](#_Toc38561926)

[11.2. Monitoring 33](#_Toc38561927)

[11.3. Source data 33](#_Toc38561928)

[11.4. Deviations or serious breaches 34](#_Toc38561929)

[11.5. Audits and inspections 34](#_Toc38561930)

[12. Ethics 34](#_Toc38561931)

[12.1. Compliance to the protocol, GCP and regulations 34](#_Toc38561932)

[12.2. Ethical review of the study 34](#_Toc38561933)

[12.3. Procedure for obtaining informed consent 34](#_Toc38561934)

[12.4. Data protection 35](#_Toc38561935)

[12.5. Insurances 35](#_Toc38561936)

[13. Substantial changes to the study 35](#_Toc38561937)

[14. Collection, handling, and archiving data 36](#_Toc38561938)

[14.1. Case Report Form (Forskningspersonsformulär) 36](#_Toc38561939)

[15. Notification of study completion, reporting, and publication 36](#_Toc38561940)

[16. References 37](#_Toc38561941)

[17. Attachments 39](#_Toc38561942)

# Signature page

**Sponsor/Coordinating Investigator**

I am responsible for ensuring that this protocol includes all essential information to be able to conduct this study. I will submit the protocol and all other important study-related information to the responsible investigator(s) so that they can conduct the study correctly. I am aware that it is my responsibility to hold the staff members who work with this study informed and trained.

| Sponsor/Coordinating Investigator’s signature Date |
| --- |

Printed name

**Principal Investigator**

I have read this protocol and agree that it includes all essential information to be able to conduct the study. By signing my name below, I agree to conduct the study in compliance with this protocol, the Declaration of Helsinki, ICH GCP (Good Clinical Practice) guidelines and the current national and international regulations governing the conduct of this clinical trial.

I will submit this protocol and all other important study-related information to the staff members and investigators who participate in this study, so that they can conduct the study correctly. I am aware of my responsibility to continuously keep the staff members and investigators who work with this study informed and trained.

| I am aware that quality control of this study will be performed in the form of monitoring, audit, and possibly inspection. |
| --- |
| Principal Investigator’s signature Date |

Printed name

# Contact information

| **Role** |  |
| --- | --- |
| Sponsor/Coordinating Investigator | Andreas Josefsson  Assistant Professor, MD, PhD  Norrlands Univeristy Hospital  Region Västerbotten  Insitute of Surgery and Perioperative Science, Dept. of Urology, University of Umeå  Försörjningvägen 1  TRC, M31, By 6M  90185 Umeå, Sweden  Phone: +46 70 3805395  e-mail: [Andreas.josefsson@umu.se](mailto:Andreas.josefsson@umu.se) |
| Principal Investigator | Andreas Josefsson (See above) |
| Clinical monitoring organization | Coordinated by Kliniskt forskningscentrum Umeå |
| Steering group | Chair  Assistant Professor Andreas Josefsson (Umeå)  Vice chair  Associate Professor Karin Welén (Gothenburg)  Senior Consultant in Oncology Camilla Thellenberg Karlsson (Umeå)  Professor Clas Ahlm (Umeå)  Professor Magnus Gisslén (Gothenburg)  Associate Professor Johan Stranne (Gothenburg)  PhD Eva Freyhult (Uppsala)  Professor Anders Bjartell (Malmö)  Professor Anna Nilsson (Malmö)  Professor Olof Akre (Stockholm)  Associate Professor Anna Jonsson-Henningsson (Jönköping)  Associate Professor David Robinsson (Jönköping)  Associate Professor Anna Bill-Axelson (Uppsala)  Associate Professor Karlis Pauksen (Uppsala)  MD/PhD Johan Styrke (Sundsvall)  MD Johanna Repo (Sundsvall)  MD/PhD Magnus Wagenius (Helsingborg)  MD Cecilia Rydén (Helsingborg)  MD/PhD Sara Cajander (Örebro)  Professor Ole Fröbert (Örebro)  MD/PhD Åse Östholm-Balkhed (Linköping)  MD/PhD Katarina Niward (Linköping) |
| Data Safety and Monitoring Board | Prof Jan-Erik Damber (Urology, Gothenburg)  Ass Prof Jan Adolfsson  (SBU)  Prof Lars Hagberg  (Infection medicine, Gothenburg)  Ass Prof Martin Eklund  (KI; Statistician)  Prof Annika Bergquist  (KI; Swedish research Council)  Helene Seeman-Lodding (GU; Intensive care) |

# List of used acronyms and abbreviations

| **Abbreviation** | **Term/Explanation** |
| --- | --- |
| ACE2 | Angiotensin-converting enzyme 2 |
| AE | Adverse Event = any untoward medical occurrence |
| AR | Adverse Reaction = adverse event, that is each unfavorable and unexpected reaction to a study treatment, regardless of dose |
| CNS | Central nervous system |
| CRF | Case Report Form |
| DSMB | Data Safety Monitoring Board |
| DSUR | Development Safety Update Report = annual safety report |
| EPM | Etikprövningsmyndigheten (English: Swedish Ethical Review Authority) |
| GCP | Good Clinical Practice |
| ICH | International Council for Harmonization |
| ICU | Intensive care unit |
| ITT | Intention-to-treat = including all data from all subjects who have participated in the study |
| LVFS | Läkemedelsverkets författningssamling (English: Swedish Medical Products Agency’s statutes) |
| MPA | Swedish Medical Products Agency |
| PCR | Polymerase chain reaction |
| PP | Per Protocol analysis = including only data from subjects who have completed the study completely in accordance with the protocol, with no deviations from the protocol |
| SAE | Serious Adverse Event = serious untoward medical occurrence |
| SPC or SmPC | Summary of Product Characteristics |
| SUSAR | Suspected Unexpected Serious Adverse Reaction |
| TMPRSS2 | Transmembrane protease, serine 2 |

# Synopsis

| EudraCT number: | 2020-002027-10 |
| --- | --- |
| Title: | A prospective, multicenter, randomized PHASE II clinical trial of enzalutamide treatment to decrease the morbidity in patients with Corona virus disease 2019 (COVID-19). |
| Study code: | COVIDENZA |
| Short background/ Rationale/Aim: | COVID-19 is a disease with high rate of morbidity if symptomatic. There is a great need of treatments to decrease the symptoms, time at hospital and need of intubated mechanical ventilation. The majority of patients needing intensive care are men. This may be due to the androgen regulation of TMPRSS2 or ACE2, necessary for virus internalization, or immune response. Enzalutamide is an antiandrogen inhibiting the activation of androgen receptors and its downstream events such as gene expression or immune cell activity. The aim of this trial is to evaluate a possible beneficial effect of short-term enzalutamide treatment of COVID-19 patients. |
| Study objectives: | Primary objective:   - Clinical status as assessed by the 7-point ordinal scale up to 30 days after inclusion   Secondary objectives:   - Safety evaluation, as measured by AEs, Adverse Reactions (ARs), SAEs, Serious ARs (SARs) - Duration of supplemental oxygen (days) - Need of mechanical ventilation - Laboratory assessment day 0, 2, 4 and 6 - Virus load assessment day 0, 2, 4 and 6 - Hospital stay (days) - Re-admission to hospital due to rebound COVID-19 - Mortality at 6 months - Hormonal status at 6 months - Serological immunity for COVID-19 at 6 months |
| Study design: | The trial is a non-blinded randomized multicenter clinical trial designed to compare the outcomes between arm 1 (experimental arm) and arm 2 (control arm) by the ratio 2:1.  Arm (1) (experimental arm):  • Enzalutamide 160mg once daily (4x40mg tablets) for 5 days + Standard of care  Arm (2) (control arm)  • Standard of care |
| Study population: | Patients with COVID-19 with mild to severe disease of the age of 50 or above. |
| Number of subjects: | N=45 (explorative phase)  N= Up to 100 (prolongation phase)  N= Up to 600 (full trial) |
| Inclusion criteria: | - Positive COVID-19 test - Mild to severe symptoms of COVID-19 - Recent hospitalization due to COVID-19 (0-3 days) - WHO performance status 0-3 - Age above or equal to 50 years - Can understand all the requirements of the study, provide informed consent, and provide authorization of use and disclosure of personal health information. - Estimated expected survival of 1 year (excluding symptoms due to COVID-19) |
| Exclusion criteria:   - Severe allergy to Enzalutamide - Pregnant or breast-feeding women - Need of immediate mechanical ventilation - Current hormonal treatment for prostate or breast cancer (finasteride and dutasteride are allowed) - Treatment for HIV - Treatment with warfarin or Clopidogrel - Treatment with immunosuppressive agents. Allowed exceptions are: equivalent medication to prednisolone 10 mg/day or low dose methotrexate 15 mg/week. Any treatment initiated as standard of care for COVID-19 is allowed. - Severe immunosuppressive disease - Stroke in medical history - Epileptic seizure in medical history - Other serious illness or medical condition - Current symptomatic unstable cardiovascular disease | |
| Investigational product(s), dosage, administration: | Enzalutamide film-coated tablets 4 x 40 mg (160mg) once daily for 5 days, per oral administration |
| Study endpoints: | Primary endpoints (variable):  **Time to need of mechanical ventilation or death**, and **time to discharge** as assessed by the 7-point ordinal scale up to 30 days after inclusion,  1) Not hospitalized, no limitations on activities.  2) Not hospitalized, limitation on activities;  3) Hospitalized, not requiring supplemental oxygen;  4) Hospitalized, requiring supplemental oxygen;  5) Hospitalized, on non-invasive ventilation or high flow oxygen devices; 6) Hospitalized, on invasive mechanical ventilation or extracorporeal membrane oxygenation (ECMO);  7) Death;  Secondary endpoint(s) (variables):  Safety evaluation, as measured by AEs, Adverse Reactions (ARs), SAEs, Serious ARs (SARs) :  30 days evaluations  Duration of supplemental oxygen (days):  Total days of extra oxygen.  Need of mechanical ventilation:  Evaluated for 30 days and after 6 months.  Laboratory assessment:  Hb, LPK, B-lymphocytes, CRP, IL-6, ASAT, ALAT, ALP, creatinine, D-dimer  Virus load assessment:  PCR based SARS-CoV-2 measurement from upper respiratory tract  Hospital stay (days)  Evaluated for 30 days and after 6 months  Re-admission to hospital due to rebound COVID-19  Evaluated for 30 days and after 6 months  Mortality at 6 months  Death due to any cause  Hormonal status at 6 months  FSH, LH, SHBG, testosterone and estrogen levels |
| Study period: | Q2 2020-Q2 2022 |

# Background and rationale

# SUMMARY

COVID-19 is a disease with high rate of morbidity if symptomatic. There is a great need of treatments to decrease the symptoms, time at hospital and need of intubated mechanical ventilation. This randomized trial will evaluate the effect of short-term enzalutamide treatment to decrease the need of worsening of the disease.

# Background

The pandemic outbreak of Sars-CoV-2 leads to high mortality and a huge burden for the health care system. To date over 2 million people have been confirmed with COVID-19 and more than 133,000 deaths have occurred (April 15^th^, Worldometers.info). Global epidemiological data shows an over-representation of men among those more severely affected and among deaths. The published data from Sweden supports this, despite showing equal risk of being diagnosed with COVID-19 for men and women, there is an increased risk of need for intensive care and risk of death for men compared to women (Folkhälsomyndigheten and SIR – intensivvårdsregistret). Of the patients in need of intensive care, about 75% are men (765 men vs 259 women (April 17^th^, Folkhälsomyndigheten).

The reason for this discriminating action of Sars-CoV-2 has, so far, not been explained. Smoking, which has been identified as a contributing risk factor for more severe disease, may be more frequent among men in some countries but in Sweden the smoking habits among men and women are similar (16% smokers among men, and 14% among women. Metabolic syndrome, conferring risk factors such as high blood pressure, obesity, and diabetes (type 2), is more frequent among men in Sweden but the difference is rather small (approximately 24% of men and 19% of women), which is not likely to explain the large difference in the response to the Sars-CoV-2 virus. However, one obvious difference between men and women is the testosterone level. A recent epidemiology study from Italy supports the notion that testosterone may influence SARS-COV-2 infection showing that prostate cancer patients on medical castration was much rarely infected compared to other male cancer patients (Montopoli et al. Ann Oncol. 2020).

It has recently been demonstrated that the Sars-CoV-2 virus employs the transmembrane protease, serine 2 (TMPRSS2) for S protein priming necessary for internalization into the cells after binding to the angiotensin-converting enzyme 2 (ACE2), and a TMPRSS2 inhibitor blocked virus entry into lung cells in vitro (Hoffman et al. Cell 2020). In a TMPRSS2 knockout mouse model, mice infected with the H1N1 influenza virus (which also uses the TMPRSS2 protein for its entrance) displayed less infection and an attenuated disease course with less lung pathology, weight loss, and mortality, compared to wild-type control mice (Hatesuer *et al*. PLoS Pathog 2013).

TMPRSS2 has been shown to be upregulated in prostate cancer compared to normal prostate epithelium and is regulated by the androgen receptor through an androgen responsive element located within an enhancer 13 kb upstream of the transcription start site (Clinckemalie *et al*. Mol Endocrinol 2013). Androgen receptors are expressed in the epithelium of the respiratory tract in humans, and it has been shown that androgen upregulated the TMPRSS2 transcript over two-fold in lung adenocarcinoma cell line (Mikkonen *et al*. Mol Cell Endocrinol 2010). Whether inhibition of androgen signaling decreases normal TMPRSS2 expression levels in not known.

In addition to regulatory effects on TMPRSS2, inhibition of androgen signaling may also affect the expression of ACE2, although the mechanism for this is unknown. ACE2 has been shown to be more expressed in airway epithelium in men than women (Muus et al. BioRxiv, 2020), and induction of ACE2 has been demonstrated in cardiac cells and in alveolar epithelium (Dalpiaz et al PLoS One, 2015 and Ghazizade et al. BioRxiv 2020). Preliminary data also show a downregulation of ACE2 in inferial bronchial tissue by castration in mice experiments (Chinnaiyan, University of Michigan, personal communication).

In addition to these specific mechanisms regulating virus infection, androgens affects the immune response. The general effect of testosterone is immunosuppressive, which possibly results in a too inefficient immune response to hamper the SARS-CoV-2 virus infection. It may also be that it is not the male immune system that is poor, but that the female, more estrogen-driven, that is better suited to deal with the SARS-CoV-2 virus. One of the severe features of the COVID-19 course is an over activation of the immune response in a so-called cytokine storm leading to damage of the lung tissue due to the inflammatory reactions. It has been shown that estrogen can induce regulatory T-cells to limit tissue damage (Tai et al. J Cell Physiol. 2008), and in a mouse model death from SARS-CoV was increased by inhibition of estrogen signaling (Channappanavar et al. J immunol. 2017). In the light of the fact that both women and men have higher estrogen levels after enzalutamide treatment (Tombal et al. Eur Urol. 2015 and Schwartzberg et al. Clin Cancer Res. 2017), this may contribute to a benefit from enzalutamide treatment for COVID-19 in both men and women.

# Benefit-risk evaluation

### Potential benefits for study participants

The pre-clinical studies show that TMPRSS2 down-regulation deteriorate the possibility for the SARS-CoV-2 to infect the lung cells, due to the strong regulation of TMPRSS2 by the androgen receptor the effect of enzalutamide treatment may eradicate further replication of the virus into new lung cells with in 24 hours after enzalutamide treatment. The benefit for the patients receiving enzalutamide may lead to both shorter time at hospital with less morbidity, lower risk of transfer to ICU and lower risk of death due to COVID-19. The strong overweight for men at the ICU may be due to the higher levels of testosterone, and if the risk of ICU care would diminished to the same levels as for women this will be of great benefit for the patients and the health care system. In women this mechanism may be as important and the benefit of decreasing the number of women to have increased morbidity and/or admission to ICU would be of great benefit for the patients and the health care system.

Further if enzalutamide is beneficial for the course of the disease, this may minimize the time at hospital and risk of sequelae for the individual patient due to no need for invasive mechanical ventilation.

Further this may be beneficial for other patient in need of the space limited ICU clinics and the stress on the health care system during the pandemic COVID-19 outbreak. The costs and stress for the COVID-19 pandemic is huge and any lessening of the burden can be of pivotal importance.

### Potential risks for study participants due to enzalutamide treatment

Enzalutamide is a potent androgen receptor inhibitor with effect on many organs including muscles, central nervous system (CNS), reproductive organs and the immune system. Even though enzalutamide is well tolerated and has few serious side effects there is an increased risk of some side effects related mostly to the CNS effects. The trials have been performed in men taking enzalutamide for sometimes many years and in many cases in metastatic prostate cancer.

The reported common side effects in both treated men and women include headache, amnesia, asthenia, tiredness, restless legs, anxiety and hot flushes. The more severe side effects include seizure (the incidence was 0.4% (13 of 3179 men) in the enzalutamide arm compared to 0.1% in the placebo arm of the combination of the trials). In the registration trials there was a small increased risk of ischemic heart disease of 2.5% compared to 1.4% in the placebo arm. The DSUR side effect report to the Swedish Medical product agency (DSUR_31AUG2018), states in the accumulated analysis: “Taken together, the non-clinical and clinical data suggest that enzalutamide is not associated with an increased risk of QT prolongation, ischemic heart disease, heart failure or arrhythmias”.

There are some possible interactions with other drugs due to induction of many cyp-enzymes which has led to exclusion criteria (below) and a notification to the clinics to be aware of and adjust the doses of Azitromycin and omeprazole.

The effect on the desire and potency due to androgen signaling inhibition of enzalutamide is known but is reversible. The effect by enzalutamide on the bone-homeostasis after long time treatment and breast tissue (especially on men) after a couple of months treatment, leading to osteoporosis and gynecomastia, respectively, are unlikely to be seen in the short-term treatment. Interactions of Enzalutamide with other medicinal products are mostly due to liver enzyme induction and may also be of less importance due to the short time of treatment. To minimize this risk we will exclude patients using medicinal products known to interact with the enzalutamide treatment.

For updated SPC see Fass.se (<https://www.fass.se/LIF/product?userType=0&nplId=20160310000078>)

The patients with COVID-19 disease with mild to severe symptoms may experience other side effects compared to the subject previously treated with enzalutamide.

The effect on immune system is poorly investigated and the potential risks that enzalutamide may interact with the immunological response to COVID-19 course cannot be ruled out and the first explorative part of the trial will be primarily be focusing in identifying any negative effect of the treatment on the course of the disease.

The same may be true for the CNS effect of enzalutamide that may affect the symptoms and this may lead to tiredness and anxiousness. This may be giving negative effect on the patient´s wellbeing and this may lead to a worse clinical evaluation of the patients not due to the course of the disease but due to any side effect of the enzalutamide. This can of course not be ruled out fully and even though the inclusion of laboratory parameters and virus load will be initially frequently evaluated in the first phase of the trial these symptoms may lead to more intensive treatment than needed if without enzalutamide.

Interaction of Enzalutamide with medication needed for the medical care may appear. This risk has been taken care of by excluding patients using medicine known to be affected by the enzalutamide treatment.

In summary, the side effects and interactions are well documented for enzalutamide and can be taken care of and the risk of any persistent side effect is minimized by the short-term treatment and frequent safety monitoring.

The risk of experiencing inconvenience by the study related procedures, including assessment of the clinical parameters, the clinical laboratory tests and the evaluation of hospital stay, 7-point ordinal scale and follow up after 6 months before the end of the trial.

The risk for the research personal and monitors in the trial to be exposed to the COVID-19 disease will be mitigated by having protocol for handling of any contaminated source data and/or forms. This will be handled according to suggested alternatives for below.

### Equipoise

Since the side effect profile of enzalutamide is considerably less harmful than the natural course of COVID-19 morbidity in hospitalized patients, the overall risk assessment is beneficial for testing enzalutamide to improve the outcome of COVID-19.

The possibility that enzalutamide may decrease the burden for the health care system may be indirectly more important than the effect on the individual patient, due to the limited capacity of the ICU clinic and health care personal, that leading to displacement effect for other diseases with mortality and morbidity.

The enzalutamide treatment is used in elderly patients in prostate cancer above 80+ and in this age the side effects seem to be few. If enzalutamide may be beneficial for the COVID-19, enzalutamide may be a great option to decrease the mortality in patients in elderly home with very little negative effects of the medicine.

# Study objectives

## Primary objective

The primary objective of this study is to evaluate the effect of enzalutamide treatment to the changes in morbidity and mortality in the course of COVID-19 in comparison to standard of care.

## Secondary objective(s)

The secondary objectives of this study are to evaluate the safety of enzalutamide treatment in COVID-19 patients, gender difference in the treatment efficacy, and evaluate serological immunity to COVID-19 in enzalutamide treated persons compared to standard of care.

## Primary endpoint (variable)

Primary endpoints (variables):

Time to need of mechanical ventilation or death, as a part of the clinical status as assessed by the 7-point ordinal scale up to 30 days after inclusion.

Time to discharge from hospital, as a part of the clinical status as assessed by the 7-point ordinal scale up to 30 days after inclusion

1) Not hospitalized, no limitations on activities.

2) Not hospitalized, limitation on activities;

3) Hospitalized, not requiring supplemental oxygen;

4) Hospitalized, requiring supplemental oxygen;

5) Hospitalized, on non-invasive ventilation or high flow oxygen devices;

## 6) Hospitalized, on invasive mechanical ventilation or extracorporeal membrane oxygenation (ECMO); 7) Death; Secondary endpoint (variables)

Safety evaluation, as measured by AEs, Adverse Reactions (ARs), SAEs, Serious ARs (SARs):

Frequency and type of “adverse reaction form” evaluated during 45 days.

Duration of supplemental oxygen (days):

Total days of extra oxygen.

Need of mechanical ventilation:

Evaluated for 30 days and after 6 months.

Laboratory assessment:

Hb, LPK, B-lymphocytes, CRP, IL-6, ASAT, ALAT, ALP, Krea, D-dimer

Virus load assessment:

PCR based SARS-CoV-2 measurement from upper respiratory tract

Hospital stay (days)

Evaluated for 45 days and after 6 months

Re-admission to hospital due to rebound COVID-19

Evaluated for 45 days and after 6 months

Mortality at 6 months

Death due to any cause

Hormonal status at 6 months

FSH, LH, SHBG, testosterone and estrogen levels

Serological immunity for COVID-19 at 6 months

Serum blood sample will be analyzed centrally for antibodies against Sars-CoV2.

# Study design and procedures

## Overall study design

This trial is a non-blinded, multicenter, prospective randomized PHASE II clinical trial of enzalutamide treatment to decrease the comorbidity in patients with Corona virus disease 2019 (COVID-19). The trial will be conducted in three phases: explorative phase is to identify any unexpected serious effect of enzalutamide treatment in this novel diagnosis. The second phase (prolongation) will be conducted to receive outcome data to be able to conduct power calculation for the last third phase (full trial). See more details below.

## Treatment arms

Arm (1) (experimental arm):

• Enzalutamide 160mg once daily (4x40mg tablets) + Standard of care

Arm (2) (control arm):

• Standard of care


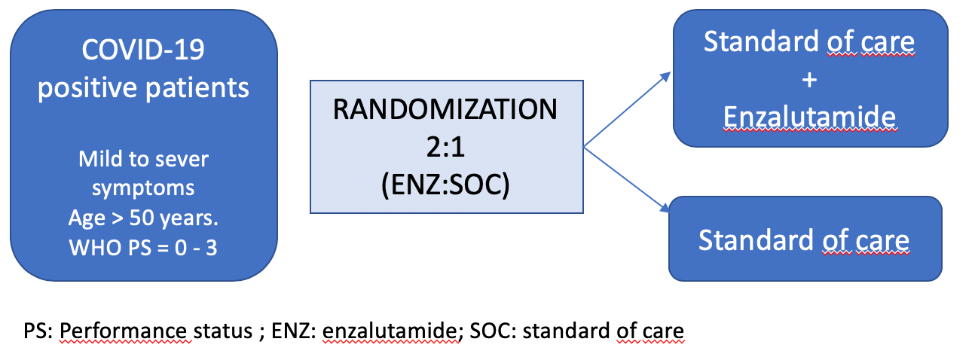


## Treatment duration

The treatment with enzalutamide will start at randomization once daily in the hospital. Patients randomized to the experimental arm will receive the investigational product for the 5 days.

## Rationale for the choice of the patient cohort

If the hypothesis is true, the most benefit would be at the time of highest virus replication in the lung cells. Due to the problem of longitudinal and multiple testing from the lower respiratory tract, it is not fully known when this phase occurs. In the phase of intensive care, the virus replication may be less prominent and it may be too late to get an effect from reducing TMPRSS2 by enzalutamide. The inclusion will therefore be for patients with confirmed COVID-19 infection having mild to severe symptoms with no need of mechanical ventilation but that may be in need of extra oxygen.

In Sweden the majority of patients have symptoms upon testing. Most patients will have mild course of the disease and will not be in need of treatment. Risk of having worsening symptoms has been shown to be in association with higher age and/or other risk factors, such as obesity, heart disease, increased number of other diagnoses. We have therefore set an age limit of at least 50 years. In frail patients, any effects on the respiratory function may be fatale but the most benefit of reducing the virus efficacy may be for patients with several other diagnoses. To balance this, we have chosen to include patients with an estimated expected survival prior to COVID-19 infection of 1 year.

Another consideration of the age limit is to exclude patients with low risk of needing intensive care and/or risk of death. Further the age limit is chosen also due to the effect on enzalutamide on hormonal effects including unknow risk in fertility and teratogenicity.

### Explorative phase:

During the first phase (explorative) 45 patients will be included. Extended testing to identify changes in virus load will be performed to identify any differences between the arms and between gender. This phase is further important to identify any serious adverse event related to enzalutamide treatment in COVID-19 patients.

During this phase the DSMB will have a weekly report from the statistician and give a report to the steering group.

If safety and possible positive effect of enzalutamide for COVID-19 from the first phase may be beneficial and if the DSMB approve to move the trial into the prolongation phase. If needed the inclusion criteria may change to include only one gender or other changes in discussion with DSMB and steering group. Any changes to the protocol will be sent in as amendment to the Swedish Medical Products Agency (MPA) and ethical committee before the inclusion can go beyond 45 patients.

### Prolongation phase:

During the second phase (prolongation) 100 patients will be included. The only change in the trial from the explorative phase is fewer blood samples will be tested at day 2 and 4.

If safety and possible positive effect of enzalutamide for COVID-19 from the prolongation phase may be beneficial the DSMB can move the trial into the full trial. Before start of the full trial a power calculation will be performed for primary end points to identify the size of the population needed to be included. In the interim analysis Cox regression will be adopted and the sample size estimate will be based on simulations. Before initiation into the next phase, inclusion criteria may change to include only one gender or other changes in discussion with DSMB and steering group. Any changes to the protocol will be sent in as amendment to the MPA and ethical committee before the inclusion can go beyond 100 patients.

### Full trial:

The trial will continue as in the prolongation phase if not otherwise decided and approved by the DSMB, Steering group, MPA and ethical committee.

## Procedures and flow chart

## Biological sampling procedures

### Handling, storage, and destruction of biological samples

Blood samples with a total volume of 10 ml to analyze Hb, LPK, Differential leukocyte count, Creatinine, CRP, ASAT, ALAT, ALP, D-dimer, IL-6 will be collected at days 0, 2, 4 and 6. Blood samples to analyze testosterone and estradiol levels will be collected at day 0 and at 6 months. Samples will be analyzed by the accredited central hospital laboratory at each site and will be destroyed immediately after analysis.
In addition, blood samples with a total volume of 15 ml for biobanking will be collected at day 0, day 6 and at 6 months. If discharged before day 6, biobanking will be performed at the day of discharge.
Five ml blood samples for analysis of Covid-19 serological immunity will be collected at 6 months and analyzed by the clinical microbiology laboratory in Umeå University Hospital, Karolinska Institute or Sahlgrenska University Hospital.

Upper respiratory swab will be collected for PCR based SARS-CoV-2 measurement at days 0, 2, 4 and 6. PCR analysis will be performed by the accredited laboratory at each site or at a central study laboratory. Residual material will be biobanked.

### Total volume of blood per subject

The total volume of blood taken from each subject during the study is maximum *100* ml.

### Biobank

Samples taken for biobanking in this study are initially handled by each site but are registered in a biobank at Biobanken Norr (no 472) and handled according to the current biobank laws and regulations. The samples are coded/pseudonymized to protect the subject´s identification. All samples and the identification/code list are stored securely and separately to prevent unauthorized persons from having access to them.

## End of Study

The study ends when the last subject has completed the last follow-up at 6 months. The study may be prematurely terminated if it appears that the treatment involved a large number of serious adverse events (SAE) or if recruitment of subjects cannot be met within reasonable time limits. If the study is prematurely terminated or suspended, the investigator should immediately inform the subjects about this and ensure appropriate treatment and follow-up. The regulatory authority should be informed as soon as possible, but no later than within 15 days.

Decisions on premature termination are taken by the sponsor.

# Subject selection

## Inclusion criteria

To be included in the study, subjects must meet the following criteria:

- - Positive COVID-19 test
  - Mild to severe symptoms of COVID-19
  - Recent hospitalization for COVID-19 symptoms (0-3 days)
  - WHO performance status 0-3
  - Age above or equal to 50 years
  - Can understand all the requirements of the study, provide informed consent, and provide authorization of use and disclosure of personal health information.
  - Estimated expected survival of 1 year (excluding symptoms due to COVID-19)
  - Women of Childbearing Capacity (WOCBC) must:

a) Comply to use of highly effective contraception methods from start of the trial until three months after the last dose of study medication. Acceptable methods according to CTFG guidelines are combined hormonal contraception (oral, dermal, intravaginal), progesterone-only hormonal contraception associated with inhibition of ovulation (oral, injectable, implantable), an intrauterine device, an intrauterine hormone-releasing system or by refraining from heterosexual intercourse during the entire period of risk.
Women of Childbearing Capacity (WOCBC) must:

b) Have a negative pregnancy test

- Male patients

a) Male patients included in the study that have fertile female partners must use adequate contraception within their relationship from start of the trial until three months after the last dose of study medication.

## Definition of COVID-19 positive test

Any clinically used test for COVID-19 disease is accepted.

## Evaluation of Severity of COVID-19 disease

### Mild

No need for extra oxygen.

### Moderate

Need of oxygen but no need of help with respiration.

No need of extra oxygen supply but saturation below 94%.

### Severe

Need of extra help with respiration.

Or No need of extra help with respiration but with saturation less than 90 with additional oxygen.

## Evaluation of comorbidity at day 0

## Other diagnoses are listed in the day 0 form. Medical treatments for any conditions are listed in the day 0 form.

## Evaluation of other risk factors at day 0

Other relevant risk factors will be evaluated in the day 0 form, including:

- Body mass index
- Smoking habits
- Gender
- Age

## Exclusion criteria

## Subjects must not be included in the study if any of the following criteria are met:

- - Severe allergy to enzalutamide
  - Pregnant or Breast-feeding women
  - Need of immediate mechanical ventilation
  - Current hormonal therapy for prostate and breast cancer (finasteride or dutasteride is allowed)
  - Treatment for HIV
  - Treatment with warfarin or Clopidogrel
  - Treatment with immunosuppressive agents. Allowed exceptions are: equivalent medication to prednisolone 10 mg/day or low dose methotrexate 15 mg/week. Any treatment initiated as standard of care for COVID-19 is allowed.
  - Severe immunosuppressive disease
  - Other serious illness or medical condition
  - Current symptomatic unstable cardiovascular disease
  - Stroke in medical history
  - Epileptic seizure in medical history

## Screening

Subject eligibility (that subjects fulfill all inclusion criteria and do not meet any exclusion criteria) is established before inclusion, treatment, or randomization. An eligibility form will be used prior to randomization in the eCRF.

## Withdrawal criteria

Subjects can discontinue their participation in the study at any time without any consequence to his/her continued treatment. The investigator/sponsor can at any time terminate the study for a subject due to, e.g., unacceptable adverse events/adverse reactions or because the subject does not follow procedures in the study protocol. If the subject discontinues the study, follow-up of this subject will be performed according to the clinic’s routine.

# Study treatments

## Description of investigational product(s)

The investigational product is enzalutamide (Xtandi^®^, manufacturer Astellas Pharma Ab), 40 mg film-coated tablets. The patients are treated for five consecutive days, 4 tablets once a day each day, during the hospitalization period. The control group is treated with standard of care.

The investigational product is provided by the manufacturer Astellas Pharma Ab. The investigational product is ordered from the hospital pharmacy according to the clinic´s normal routines and taken from the clinic´s shelf. If changes take place between wards, local routines will be adopted.

## Dose and administration

The defined dose for men in prostate cancer will be used. This means 4 film-coated tablets of 40mg each, once daily, taken orally. See also (<https://www.fass.se/LIF/product?userType=0&nplId=20160310000078>)

## Packaging, labeling, and handling of investigational products(s)

The investigational product is an approved medicinal product (Xtandi^®^) in Sweden. The package currently marketed in Sweden, 4x28 film-coated tablets in blister, is used in the trial. Xtandi^®^ is ordered from the hospital pharmacy according to the clinic´s normal routines and stored in the clinic´s shelf according to the clinic´s normal routines. No additional labelling for clinical trial is used. Discharged patients or patients transferred to ICU will not continue with enzalutamide treatment.

## Drug accountability and treatment compliance

Drug accountability will be registered everyday (day 0-4), and treatment compliance will be registered in the CRF.

## Randomization

Subjects are included/randomized consecutively as they are found to be eligible for inclusion in the study. If a subject discontinues their study participation, their subject code will not be reused and the subject will not be allowed to re-enter the study again.

The patients will be randomized by the randomization function in the REDcap (version 9.5.18) after fulfilling the checklist in the trial. Randomization will be 2:1 (enzalutamide + “standard of care”: “standard of care”). The consecutive randomization number for each list created prior to inclusion start will be arranged to enable randomization of 600 patients per site with 250 per gender.

Patients will be stratified at randomization according to:

- Centre
- Gender

The natural course of patients without treatment will be known due to ongoing studies, however as standard treatment during the pandemic is changing, the outcome of the patients in the control arm (not receiving enzalutamide) will be compared to known data at interim analysis. This will enable adjustment of the ratio if needed between the prolongation phase and the full trial.

Randomization will be performed by direct contact with the local trial nurse with access to the eCRF or by calling a specified phone helping with the randomization in the eCRF.

## Concomitant medications

Medications that are considered necessary for the safety and well-being of the subject can be given at the discretion of the investigator, unless otherwise specified as an exclusion criterion. Concomitant medications should be reported in the Case Report Form (CRF), with exception for on demand medication by topical administration.

Concomitant medication with omeprazole will need adjustment to have the clinical effect. We recommend doubling the dose. If azithromycin is indicated we recommend change to similar antibiotic.

## Destruction

Drugs not used will be disposed at the pharmacy or at the hospital for further disposal according to local regulations.

## Treatment after study end

The treatment period with investigational product in this trial is 5 days or until discharge from the hospital or need of mechanical ventilation, whichever occurs first. After day 7 there is no restriction of medical treatments or inclusion in studies for any experimental therapies. If the patient is stopping treatment of enzalutamide earlier due to any cause, there are not restrictions for after treatments.

# Methods for measurement of endpoints for clinical efficacy and safety

## Methods for measurement of endpoints for clinical efficacy

### Primary endpoint (variable)

The ordinal scale used to evaluate the primary end-point is used in other trials for COVID-19. It is based on the level of oxygen need if hospitalized and if not hospitalized if the patient has any symptoms or are without symptoms. (See below)

If the patient is at the hospital the 7-point ordinal scale form will be filled. If not hospitalized the follow up will be by phone and registered directly in the eCRF by study personal.

Clinical status as assessed by the 7-point ordinal scale up to 30 days after inclusion:

1) Not hospitalized, no limitations on activities. D

2) Not hospitalized, limitation on activities;

3) Hospitalized, not requiring supplemental oxygen;

4) Hospitalized, requiring supplemental oxygen;

5) Hospitalized, on non-invasive ventilation or high flow oxygen devices;

6) Hospitalized, on invasive mechanical ventilation or extracorporeal membrane oxygenation (ECMO);

7) Death;

### Secondary endpoints (variables)

Safety evaluation, as measured by AEs, Adverse Reactions (ARs), SAEs, Serious ARs (SARs):

Frequency and type of adverse reaction will be evaluated during the first 45 days. The responsible doctor will have to answer if the AE is related to the treatment of enzalutamide or if the patient has started any other investigational drug, and state the possible relationship to either drug

**Duration of supplemental oxygen (days):**

Total days of extra oxygen described as any additional oxygen given to the patient at any time during the day between 00-24.

Need of mechanical ventilation:

If the patient is in need of mechanical ventilation the date of this will be defined. Evaluated for 30 days and after 6 months.

**Laboratory assessment:**

The local clinical lab will be used for all laboratory evaluation. Hb, LPK, B-lymphocytes, CRP, IL-6, ASAT, ALAT, ALP, Krea, D-dimer

**Virus load assessment:**

PCR based SARS-CoV-2 measurement from upper respiratory tract.

The tests will be handled in the clinic where the patients is admitted at day 0, 2, 4 and 6. If the patient is discharged before day 6, the last test will be taken at the day of discharge.

Only the relative change of the PCR will compared for the patient and therefore the PCR will be performed at the same day after all sample have been collected at the labs used by the local clinic where the samples has been taken, or transferred to a central study laboratory

**Hospital stay (days)**

Evaluated for 45 days and after 6 months

Any hospitalization any time during any day between 00-24 will be considered as admitted to the hospital that day. At day 2, 4, 6, 7, 14, 21, 30, 45 and 6 months the form includes if the patient is admitted primarily based on COVID-19 symptoms.

**Re-admission to hospital due to rebound COVID-19**

Evaluated for 45 days and after 6 months

Any hospitalization any time during any day between 00-24 will be considered as admitted to the hospital that day. Only if the patient has been home from one day to the next it will be described as re-admission (and at least 12 hours). Any worsening symptoms considered to be a consequence from COVID-19 requiring re-hospitalization is regarded as a rebound event. Rehabilitation at hospital with stable symptoms is not regarded as a rebound event.

**Mortality at 6 months**

Death due to any cause and death date will be found by the study nurse through the medical records or by death registry.

**Hormonal status at 6 months**

The local clinical laboratory will be used to evaluate the hormonal status. FSH, LH, SHBG, testosterone and estrogen/estradiol levels

**Serological immunity for COVID-19 at 6 months**

Serum blood sample will be analyzed centrally for antibodies against Sars-CoV2.

## Methods for measurement of endpoints (variables) for clinical safety

The safety endpoint includes clinical evaluation day 0, 2, 4, 6, 7, 14, 21, 30 and 45. For days 14, 21, 30, and 45 a variation of ± 2 days is allowed. For the 6 months evaluation a variation of ± 2 weeks is allowed. The local routine, and measurement methods available, will be used to evaluate heart rate (per minutes), respiration frequency (per minutes), blood pressure (Systolic blood pressure/diastolic blood pressure), fever (the method will be noted) and saturation (the oxygen supply l/min will be noted).

# Handling of Adverse Events

## Definitions

### Adverse Event (AE)

Adverse Event (AE): Any untoward medical occurrence in a clinical investigation subject administered a medicinal product and, which does not necessarily have a causal relationship with the treatment, can be an unfavorable and unintended sign (including an abnormal laboratory discovery), symptom or disease temporally associated with the use of the medicinal (investigational) product, whether or not related to the medicinal (investigational) product.

### Adverse Reaction (AR)

In the pre-approval clinical experience with a new medicinal product or new use of a medicinal product, and particularly as the therapeutic dose(s) may not be established, all noxious and unintended reactions to the medicinal product related to any dose should be considered an adverse reaction (AR). The phrase “reaction” to a medicinal product means that the causal relationship between the medical product and an adverse event is at least a reasonable possibility; that is the relationship cannot be ruled out.

### Serious Adverse Event (SAE)

Serious adverse event (SAE): Any untoward medical occurrence that at any dose:

- results in death
- is life-threatening
- requires inpatient hospitalization or prolongation of existing hospitalization
- results in persistent or significant disability or incapacity
- results in a congenital anomaly/malformation

Medical and scientific assessment will be made to determine if an event is “serious” and whether it would prompt reporting in other situations, for example important medical events that may not be directly life-threatening or result in death or hospitalization but may compromise the study subject or may require intervention to prevent one of the other results set forth in the definitions above. These should also normally be considered as SAEs.

### Suspected Unexpected Serious Adverse Reaction (SUSAR)

SUSAR: A reaction/event that is unexpected, serious, and suspected to be caused by the treatment, i.e. adverse events that are not included in the SPC.

## Assessment of Adverse Events

### Assessment of causal relationship

The investigator is responsible for determining whether there is a causal relationship between the AE/SAE and use of the investigational product. Further in this trial the assessment will also be taken to define if there is any causal relationship to any investigational therapy in trials starting after the 7 days follow up in this trial (or after end of trial due to other reasons).

Those AEs which are suspected of having a relationship to the investigational product will be followed up until the subject has recovered or is well taken care of and on their way to good recovery (see also section 9.4, Follow-up of Adverse Events).

All AE will be categorized either as likely related, possibly related, or not related, in accordance with the definitions below:

**Likely related**: Clinical event, including abnormal results from laboratory analyses, occurring within a reasonable time after administration of the intervention/investigational product. It is unlikely that the event can be attributed to underlying disease or other medications, but is most likely caused by the investigational product and its emergence is reasonable in relationship with use of the investigational product.

**Possibly related**: Clinical event, including abnormal results from laboratory analyses, occurring within a reasonable time after administration of the intervention/investigational product. The event could be explained by the investigational product and its emergence is reasonable in relationship with use of the investigational product, but there is insufficient information to determine the relationship. The event could be explained by an underlying disease or other medications.

**Not related**: Clinical event, including abnormal results from laboratory analyses, that is not reasonably related to the use of the intervention/investigational product. The event is unlikely related to the intervention/investigational product and can be explained by other medications or underlying disease.

### Assessment of intensity

Each adverse event shall be classified by CTCAEv5.0 criterias. (<https://ctep.cancer.gov/protocolDevelopment/electronic_applications/ctc.htm#ctc_50>).

### Assessment of seriousness

The investigator is responsible for assessing the seriousness (serious or non-serious). If the incident is considered serious, this should be reported as a serious adverse event (SAE) by the investigator to the sponsor. See also section 9.3.1, Reporting of Serious Adverse Events (SAE).

## Reporting and registration of Adverse Events (AE)

From the time of signed informed consent until 45 days, for all patients in the trial, AE will be assessed. All AE that occur during the study and which are observed by the investigator/study nurse or reported by the subject will be registered in the CRF regardless of whether they are related to the investigational product or not. Assessment of causal relationship, severity, and whether the AE is considered to be an SAE or not will be done by the investigator directly in CRF/on study-specific worksheet. A study nurse will fill in information from study-specific worksheet in the eCRF. At minimum, for each AE/SAE, a description of the event is recorded (diagnosis/symptom if diagnosis is missing), start and stop dates, causal relationship, severity, if the AE is considered to be an SAE or not, measures and outcome. Due to the COVID-19 disease local routines will be adopted to secure the personal in the study in the management of the transfer from worksheet to the eCRF.

Events not reported as AE in this trial

The COVID-19 disease has a clinical course including serious illness with potential threat to the patient’s life. These parameters will be followed (see list below) and any change in these parameters will not be reported as AE or SAE.

List of parameters expected:

- Oxygen consumption
- Fever
- Need of mechanical respiratory ventilation
- Dizziness
- Hypertension
- Hypotension
- Tachycardia
- Bradycardia

### Reporting of Serious Adverse Events (SAE)

Serious adverse events (SAE) are reported to the sponsor, via fax to Kliniskt Forskningscentrum Umeå, on a special SAE form within 24 hours of the investigator (or any other study staff) being informed of the SAE. The investigator determines if the event meets the protocol definition of an SAE, assesses causal relationship and intensity of the event. All SAE are reported to the sponsor independent of causality. The sponsor decides if the SAE fulfills the SUSAR criteria, and if so, is responsible for notifying the regulatory authorities (for SUSAR timelines see section 9.3.2).

Follow-up information describing the outcome and handling of the SAE is reported, via fax to Kliniskt Forskningscentrum Umeå, as soon as this information is available. The original should be kept in the Investigator Site File.

### Reporting of Suspected Unexpected Serious Adverse Reactions (SUSAR)

Those SAE which are assessed by sponsor to be SUSAR are reported via a [CIOMS form](https://cioms.ch/wp-content/uploads/2017/05/cioms-form1.pdf) to the European Medicines Agency (EudraVigilance database) according to the specified time frames.

SUSAR that are fatal or life-threatening are reported as soon as possible and no later than 7 days after the incident has become known to the sponsor. Relevant follow-up information is sent thereafter within an additional 8 days. Other SUSAR are reported as soon as possible and no later than 15 days after they have come to the sponsor’s knowledge.

Information about SUSAR occurring during the study is compiled by the sponsor and sent out to the principal investigator at all participating centers.

## Follow-up of Adverse Events

All reported SAEs that have not been resolved by the end of the study will be followed up until the event has subsided (or disappeared), the condition has stabilized, the event is otherwise explained or the study subject is lost to follow-up. SAE occurring after trial termination must be reported if considered related to the treatment during the trial.

## Independent Data Safety and Monitoring Committee (DSMB)

An advisory committee with 4-5 members will meet with the study board every week until 45 patients have been followed for at least 3 weeks and there after based on the decision of the DSMB, but at least once monthly until 100 patients has been included and followed. DSMB shall be eligible to stop randomization for safety reasons. They will meet by video conference.

This committee will advise the steering group in recommending possible extensions of the study and changes/amendments in the protocol. The DSMB may also be asked for advice in other questions brought up by the steering board.

## Annual Safety Report (Development Safety Update Report, DSUR)

A yearly safety report will be sent to the Swedish Medical Products Agency and the Swedish Ethical Review Authority. The safety report will summarize all serious adverse events recorded during the year, as well as a summary assessment of the safety of patients and if the risk-benefit assessment has changed.

## Procedures in case of emergencies, overdose or pregnancy

The sponsor and investigator will immediately take the urgent safety measures necessary to protect the subjects from immediate danger. The sponsor will inform the Swedish Medical Products Agency and EPM as soon as possible about the urgent safety measures taken by the investigator or sponsor.

If a study subject who participates in the trial becomes pregnant, this person must be followed up until the birth has taken place. If the fetus/child has any congenital malformation, this must be reported as a serious adverse event or side effect (SAE).

## Reference Safety Information

SPC for Enzalutamid will be used for reference safety information regarding assessment of whether an adverse event/incident (AE) is expected or not.

For updated SPC see Fass.se (<https://www.fass.se/LIF/product?userType=0&nplId=20160310000078>)

## Enzalutamide safety information in summary

Enzalutamide (Xtandi^®^, Astellas Pharma, film-coated tablet) is an androgen receptor inhibitor used in patients with prostate cancer and has a beneficial tolerance with few side effects in men and has also been tested in breast cancer patients in a few clinical trials with no more side effects compared to known side effects. Over 12000 men (2018) have been given enzalutamide in different clinical trials and it has been used as second and third-line treatment for prostate cancer in Europe and US since 2012.

For detailed information we refer to [www.fass.se](http://www.fass.se)

# Statistics

## Analysis population

Two efficacy analysis sets will be used: the ‘all patients treated’ set which will be analyzed according to the intention-to-treat (ITT) principle and it is therefore called the ITT set and the ‘per protocol’ (PP) set. The primary and secondary statistical analysis will be based on the ITT set. In addition, an analysis of the efficacy variables will also be carried out on the PP set in order to detect any sensitivity in the results obtained from the ITT analysis. At the clean file meeting it will be decided whether a patient will be included in the ITT and/or PP population. Also, a safety analysis set will be used for the assessment of the safety variables.

***The ITT analysis set***
All randomized subjects will be included in the ITT analysis set.

***UThe PP analysis set***
All subjects from the ITT set with no major protocol deviations and who have completed at least 8 cycles of treatment according to the protocol.

***USafety analysis set***
All randomized subjects will be included in the safety analysis set. This set is identical to the ITT set.

- 1. Statistical analyses

### 10.2.1 Statistical methods

### Summary statistics

Clinical parameters and laboratory assessments will be summarized in total and stratified for gender, center and study arm.

Continuous variables will be summarized using standard statistical measures, i.e. the number of observations, number of missing observations, mean, standard deviation, minimum, 1st quartile, median, 3rd quartile and maximum. Categorical variables will be summarized in frequency tables.

### Primary objective

Time to discharge will be compared using the ordinal scale, between study arms using Cox regression adjusting for site, age and gender.

Time to need of mechanical ventilation or death will be compared using the ordinal scale, between study arms using Cox regression adjusting for site and gender.

### Secondary objectives

Frequencies of Adverse events will be compared between the two arms using Fisher’s exact test.

Duration of supplemental oxygen (days) will compared between study arms using linear regression adjusted for gender and site.

Time to need of mechanical ventilation will be compared using the ordinal scale, between study arms using Cox regression. And the frequency of need of mechanical ventilation in the two arms will be tested using Fisher’s exact test.

Laboratory assessments at each time of measurement will be compared to baseline and extreme value and differences between study arms evaluated using linear or generalized linear regression adjusting for site, age and gender.

The change in viral load on day 2, 4 and 6 as compared to baseline will be compared between study arms. Also, the maximum decrease in viral load will be calculated, as the difference between observed value and maximal observed value, and compared between study arms. The analyses will be performed using linear regression adjusting for site and gender.

Total hospital stay (days) evaluated at 30 days and 6 months will be compared between groups using linear regression adjusting for site, age and gender.

Frequency of re-admission to hospital due to rebound COVID-19 will be compared between study arms using Fisher’s exact test.

Time to death will be compared between study arms using Cox regression adjusting for site and gender.

Hormonal status at 6 months will be compared to normal reference values and compared between study arms.

Frequency of serological immunity for Covid-19 will be compared between the study arms using Fisher’s exact test.

### Drop-outs

In all survival analyses drop-out will be censored at time of drop-out. For all other analyses drop-outs will be excluded from the analyses.

- 1. Adjustment of significance and confidence interval

Indicate possible tests for multiple comparisons. Adjustment should always be considered for multiple primary outcomes. Specify details of any adjustment procedures or provide an explanation for why justification is not considered necessary.

Multiple testing correction will be performed using Bonferroni’s methods to adjust for having two primary outcomes.

- 1. Sample size calculations

The sample size of the full study will be determined based on data from the prolongation study.

- 1. Interim analysis (if relevant)

The primary outcomes will be evaluated after prolongation phase.

# Quality Control and Quality Assurance

## Quality Assurance and Sponsor oversight

It is the investigators responsibility that the CRFs are reviewed for completeness and accuracy. It is also the investigators responsibility to ensure compliance with GCP and all applicable regulatory requirements. Regulatory agencies may conduct a regulatory inspection of this trial. Such audits/inspections can occur at any time during or after completion of the trial. If an audit or inspection occurs, the investigator and institution agree to allow the audit/inspection.

## Monitoring

The study will be monitored by an independent monitor before the study begins, during the study conduct, and after the study has been completed, so as to ensure that the study is carried out according to the protocol and that data is collected, documented, and reported according to ICH-GCP and applicable ethical and regulatory requirements. Monitoring is performed as per the study’s monitoring plan and is intended to ensure that the subject’s rights, safety, and well-being are met as well as data in the CRF are complete, correct, and consistent with the source data.

To verify this, the study will be monitored in a systematic, prioritized, risk-based approach consistent with the demands of the trial and site activity. If any misconduct or neglect would be notified the sponsor and investigator will be notified for corrective and preventive actions.

The investigator and the head of the medical institution (where applicable) agrees to allow the monitor access to all relevant documents.

Data monitoring will be performed regularly, by either monitoring at site at a computer not in the same wards as COVID-19 positive patients, or by phone and or video conference with the monitor with direct access to the database with the eCRF and personal at the hospital.
Monitoring details will be specified in the study monitoring plan.

## Source data

The investigator must keep source documents for each subject in the study. A document describing what has been classified as source data in the study should be included in the Investigator Site File (ISF). The investigator must ensure that all source documents are accessible for monitoring and other quality control activities.

Source data is defined before study start at each individual site.

The investigator must ensure that all source documents are accessible for monitoring and other quality control activities. The monitor will have access to medical records and source data after secrecy agreement have been signed by the responsible party at the site as well as by the monitor. Subjects have provided consent by signing the Subject Information and Informed Consent form where this is specified.

## Deviations or serious breaches

Serious breaches and deviations from the study protocol, GCP and other regulations that significantly and directly affects, or with high likelihood could affect, the subjects in Sweden or the scientific value of the study, shall be immediately reported within 7 days (from knowledge) to the Swedish Medical Products Agency. It is the sponsor’s responsibility to judge the consequences of deviations that have occurred, and thus also to decide whether the Swedish Medical Products Agency should be informed.

Minor deviations that do not affect subjects’ integrity or safety, nor significantly affect the study’s scientific value, are documented in the study documentation of the principal investigator and the sponsor.

## Audits and inspections

Authorized representatives for the sponsor and Competent Authorities (CA) may carry out audits or inspections at the study site, including source data verification. The investigator must ensure that all source documents are available for audits and inspections. The purpose of an audit or inspection is to systematically and independently review all study-related activities and documents, so as to determine whether these activities were performed, registered, analyzed and reported correctly according to protocol, Good Clinical Practice (GCP) and applicable regulations.

# Ethics

## Compliance to the protocol, GCP and regulations

The study will be performed in compliance with the study protocol, the Declaration of Helsinki, ICH-GCP (Good Clinical Practice) guidelines and current national and international regulations governing this clinical trial. This is to ensure the safety and integrity of the study subjects as well as the quality of the data collected.

## Ethical review of the study

The final study protocol for clinical trials must be approved, as a part of the application for a permit for clinical trials, by both the Swedish Ethical Review Authority (Etikprövningsmyndigheten, EPM) and the Swedish Medical Products Agency before the trial can be conducted. The final version of the informed consent form and other information provided to subjects, must be approved or given a written positive opinion by EPM. EPM and the Swedish Medical Products Agency must be informed of any changes in the study protocol in accordance with current requirements.

## Procedure for obtaining informed consent

The principal investigator at each site shall ensure that the subject is given full and adequate oral and written information about the study, its purpose, any risks and benefits as well as inclusion and exclusion criteria. Subjects must also be informed that they are free to discontinue their participation in the study at any time without having to provide a reason. Subjects should be given the opportunity to ask questions and be allowed time to consider the provided information. If the person chooses to participate, both the subject and the investigator shall sign the informed consent form. A copy of the subject information as well as the informed consent form shall be provided to the subject. Due to the risk of contamination, the signed informed consent will be photographed and assessed as source data. Local variations on this procedure are allowed. The subject’s signed and dated informed consent must be obtained before performing any study-specific activity in the study. Each subject who participated in the study will be identified by a subject number on a subject identification list. The subject agrees that monitors, auditors, and inspectors may have access to their medical records and other source data. If new information is added to the study, the subject has the right to reconsider whether he/she will continue their participation.

## Data protection

If any part of the data is handled by any other organization, inside or outside the EU, appropriate agreements and/or other documentation will be established, to ensure that the data processing is performed in accordance with the provisions of the General Data Protection Regulation (GDPR) and other relevant legislation, before any data transfer takes place.

The content of the informed consent form complies with relevant integrity and data protection legislation. In the subject information and the informed consent form, the subject will be given complete information about how collection, use and publication of their study data will take place. The subject information and the informed consent form will explain how study data are stored to maintain confidentiality in accordance with national data legislation. Data collected in the eCRF REDCap will be stored at secure servers at Umeå University. All information processed by the sponsor will be pseudonymized and identified with study ID.

The informed consent form will also explain that for verification of the data, authorized representatives of the sponsor, as well as relevant authority, may require access to parts of medical records or study records that are relevant to the study, including the subject’s medical history.

## Insurances

Study subjects are insured through Swedish patient insurance as well as the Swedish pharmaceutical insurance.

# Substantial changes to the study

Substantial changes to the signed study protocol are only possible through approved protocol amendments and by agreement from all responsible persons. Information on non-substantial changes should be clearly noted in the amended protocol.

In the event that substantial changes to the protocol (e.g., changing of the main objective, primary or secondary variables, method to measure the primary variable, changing of the investigational product or dosage) will be made during the course of the study, approval from the Swedish Ethical Review Authority (Etikprövningsmyndigheten, EPM) as well as the Swedish Medical Products Agency (Läkemedelsverket) shall be obtained before any changes are implemented. A change that concerns a new site, new investigator or a new study patient information sheet shall only be approved by EPM.

Non-substantial changes will be recorded and later entered in documentation that is submitted, for example in any subsequent notifications of a substantial change or in connection with End of Trial reporting.

The investigator must not make any deviation from or change of the protocol, except if necessary to eliminate an immediate risk to the study subjects.

# Collection, handling, and archiving data

Subjects who participate in the study are coded with a specific study identification number. All subjects are registered in a subject identification list (subject enrolment and identification list) that connects the subject’s name and personal number with a study identification number.

All data will be registered, managed, and stored in a manner that enables correct reporting, interpretation, and verification. The complete Trial Master File, as well as source documents, will be archived for at least *15* years after the study is completed. Source data in the medical records system is stored and archived in accordance with the respective hospital regulations.

## Case Report Form (Forskningspersonsformulär)

An electronic Case Report Form (eCRF), REDCap, is used for data collection. The investigator must ensure that data is registered and any corrections in the CRF are made as stated in the study protocol and in accordance with the instructions. The investigator must ensure that the registered data is correct, complete, and that reporting takes place according to the timelines that have been predefined and agreed. The investigator signs the completed CRF. A copy of the completed CRF will be archived at the study site.

If an examination/test is not performed and data does not exist, ND (Not done) or NK (Not known) is marked. If the question is irrelevant NA (Not applicable) is written. Corrections in the paper CRF/working sheets are done by striking out the incorrect information and adding the correct information next to the incorrect information, signing, and dating the correction.

# Notification of study completion, reporting, and publication

The Swedish Medical Products Agency shall be informed of the study’s completion at latest 90 days after study end, through submission of a ”Declaration of End of Trial Notification” form.

Within one year after the study is completed, the results shall be analyzed, a clinical study report with individual data shall be prepared, and the study results shall also be reported in the EudraCT database.

# References

Channappanavar, R., Fett, C., Mack, M., Ten Eyck, P. P., Meyerholz, D. K., & Perlman, S. (2017). Sex-Based Differences in Susceptibility to Severe Acute Respiratory Syndrome Coronavirus Infection. J Immunol, 198(10), 4046-4053. doi:10.4049/jimmunol.1601896

Clinckemalie, L., Spans, L., Dubois, V., Laurent, M., Helsen, C., Joniau, S., & Claessens, F. (2013). Androgen regulation of the TMPRSS2 gene and the effect of a SNP in an androgen response element. Mol Endocrinol, 27(12), 2028-2040. doi:10.1210/me.2013-1098

Dalpiaz, P. L., Lamas, A. Z., Caliman, I. F., Ribeiro, R. F., Jr., Abreu, G. R., Moyses, M. R., . . . Bissoli, N. S. (2015). Sex Hormones Promote Opposite Effects on ACE and ACE2 Activity, Hypertrophy and Cardiac Contractility in Spontaneously Hypertensive Rats. PLoS One, 10(5), e0127515. doi:10.1371/journal.pone.0127515

Ghazizadeh, Z., Majd, H., Richter, M., Samuel, R., Zekavat, S. M., Asgharian, H., . . . Fattahi, F. (2020). Androgen Regulates SARS-CoV-2 Receptor Levels and Is Associated with Severe COVID-19 Symptoms in Men. BioRxiv, doi.org/10.1101/2020.05.12.091082.

Hatesuer, B., Bertram, S., Mehnert, N., Bahgat, M. M., Nelson, P. S., Pohlmann, S., & Schughart, K. (2013). Tmprss2 is essential for influenza H1N1 virus pathogenesis in mice. PLoS Pathog, 9(12), e1003774. doi:10.1371/journal.ppat.1003774

Hoffmann, M., Kleine-Weber, H., Schroeder, S., Kruger, N., Herrler, T., Erichsen, S., . . . Pohlmann, S. (2020). SARS-CoV-2 Cell Entry Depends on ACE2 and TMPRSS2 and Is Blocked by a Clinically Proven Protease Inhibitor. Cell. doi:10.1016/j.cell.2020.02.052

Mikkonen, L., Pihlajamaa, P., Sahu, B., Zhang, F. P., & Janne, O. A. (2010). Androgen receptor and androgen-dependent gene expression in lung. Mol Cell Endocrinol, 317(1-2), 14-24. doi:10.1016/j.mce.2009.12.022

Montopoli, M., Zumerle, S., Vettor, R., Rugge, M., Zorzi, M., Catapano, C. V., . . . Alimonti, A. (2020). Androgen-deprivation therapies for prostate cancer and risk of infection by SARS-CoV-2: a population-based study (n=4532). Ann Oncol. doi:10.1016/j.annonc.2020.04.479

Muus, C., Luecken, M., Eraslan, G., Waghray, A., Heimberg, G., Sikkerna, L., & al., e. (2020). Integrated analyses of single-cell atlases reveal age, gender, and smoking status associationswith cell type-specific expression of mediators of SARS-CoV-2 viral entry and highlights inflammatory programs in putative target cells. BioRxiv, doi.org/10.1101/2020.04.19.049254.

Schwartzberg, L. S., Yardley, D. A., Elias, A. D., Patel, M., LoRusso, P., Burris, H. A., . . . Traina, T. A. (2017). A Phase I/Ib Study of Enzalutamide Alone and in Combination with Endocrine Therapies in Women with Advanced Breast Cancer. Clin Cancer Res, 23(15), 4046-4054. doi:10.1158/1078-0432.CCR-16-2339

Tai, P., Wang, J., Jin, H., Song, X., Yan, J., Kang, Y., . . . Wang, B. (2008). Induction of regulatory T cells by physiological level estrogen. J Cell Physiol, 214(2), 456-464. doi:10.1002/jcp.21221

Tombal, B., Borre, M., Rathenborg, P., Werbrouck, P., Van Poppel, H., Heidenreich, A., . . . Smith, M. R. (2015). Long-term Efficacy and Safety of Enzalutamide Monotherapy in Hormone-naive Prostate Cancer: 1- and 2-Year Open-label Follow-up Results. Eur Urol, 68(5), 787-794. doi:10.1016/j.eururo.2015.01.027

# Attachments

1. Current Swedish Summary of Product Characteristics (SPC) of Xtandi^®^ 40 mg film-coated tablets (FASS)
